# Central nervous system progression in advanced non–small cell lung cancer patients with EGFR mutations in response to first-line treatment with two EGFR-TKIs, gefitinib and erlotinib: a comparative study

**DOI:** 10.1186/s12885-017-3165-0

**Published:** 2017-04-04

**Authors:** Meng-Xia Li, Hao He, Zhi-Hua Ruan, Yu-Xi Zhu, Rong-Qing Li, Xiao He, Bao-Hua Lan, Zhi-Min Zhang, Guo-Dong Liu, Hua-Liang Xiao, Yan Wu, Bo Zhu, Ge Wang, Zhen-Zhou Yang

**Affiliations:** 1 Cancer Center, Research Institute of Surgery, Daping Hospital, Third Military Medical University, 10 Changjiang Zhilu, Daping Yuzhong District, Chongqing, 400042 People’s Republic of China; 2 Institute of Cancer, Xinqiao Hospital, Third Military Medical University, Chongqing, 400037 People’s Republic of China; 3 Department of Oncology, Southwest Hospital, Third Military Medical University, Chongqing, 400037 People’s Republic of China; 4 Department of Oncology, First Affiliated Hospital of Chongqing Medical University, Chongqing, 400016 People’s Republic of China; 5 Department of Radiation Oncology, First Affiliated Hospital of Kunming Medical University, Kunming, 650032 Yunnan People’s Republic of China; 6 Eighth Department, Research Institute of Surgery, Daping Hospital, Third Military Medical University, Chongqing, 400042 People’s Republic of China; 7 Department of Pathology, Research Institute of Surgery, Daping Hospital, Third Military Medical University, Chongqing, 400042 People’s Republic of China

**Keywords:** EGFR-TKIs, Erlotinib, Gefitinib, NSCLC, EGFR mutation

## Abstract

**Background:**

Central nervous system (CNS) brain metastasis of advanced non-small cell lung cancer (NSCLC) patients confers a worse quality of life and prognosis. The efficacy comparison of two first-generation epidermal growth factor receptor (EGFR) inhibitors erlotinib or gefitinib as first-line treatment for CNS metastasis NSCLC patients with EGFR-sensitizing mutations is yet to be elucidated.

**Methods:**

A retrospective analysis was done on cerebral metastasis rate after erlotinib or gefitinib as first-line treatment for advanced NSCLC patients with EGFR-sensitizing mutations. Time to neurological progression (nTTP) and median progression-free survival (mPFS) were calculated.

**Results:**

The study involved 279 patients (erlotinib group: 108, gefitinib group: 171). After a median follow-up of 22 months, 27 patients (25%) in the erlotinib group and 60 patients (35.1%) in the gefitinib group showed CNS progression. The HR of CNS progression for erlotinib versus gefitinib was 0.695 [95% confidence interval (CI), 0.406–1.190], suggesting a risk reduction of 30.5% although not achieving statistical significance. The 6-, 12- and 18-month cumulative CNS progression rates were 0.9, 3.7 and 12% for erlotinib compared with corresponding rates of 5.8, 9.4 and 17% for gefitinib (*P* = 0.181). However, for those patients with preexisting brain metastases prior to EGFR-TKI treatment, erlotinib as first line treatment significantly extended the median nTTP in comparison to gefitinib (30 months vs 15.8 months, *p* = 0.024).

**Conclusions:**

Our data show that nTTP can be effectively extended in preexisting brain metastases patients with EGFR-sensitizing mutations initially treated with erlotinib compared with gefitinib. If confirmed, our results indicate that erlotinib may play an important role in controlling CNS progression from EGFR mutation-positive NSCLC.

**Electronic supplementary material:**

The online version of this article (doi:10.1186/s12885-017-3165-0) contains supplementary material, which is available to authorized users.

## Background

Brain metastases occur in about 10% of NSCLC patients at the initial diagnosis and in about 40–55% of patients during the entire course of the disease. In particular, EGFR mutations show a strong association with the risk of brain metastases at the initial time of diagnosis and follow up in lung adenocarcinoma patients [1–4]. CNS brain metastasis confers a worse quality of life and prognosis [5]. Conventional chemotherapy drugs are difficult to pass through the blood brain barrier. NSCLC patients with multiple brain metastases treated with whole brain radiotherapy (WBRT) have an overall survival (OS) of 3–6 months and a 1-year survival rate of only 10–20% [6]. Concurrent chemotherapy with WBRT can improve the effective rate but cannot prolong OS of NSCLC brain metastases patients [7]. With continuous improvement of systemic treatment for NSCLC, possible therapeutic strategies for preventing and controlling brain metastasis to improve overall disease control and quality of life becomes more critical.

The outcomes from multiple prospective phase III clinical trials have shown significantly better clinical efficacy in EGFR mutant advanced NSCLC patients initially treated with first- generation EGFR inhibitors (gefitinib or erlotinib) compared with upfront chemotherapy, with an objective response rate (ORR) of 71–83% and PFS of 9–13 months [8, 9]. Recently published data showed that erlotinib and gefitinib could efficiently pass through the blood brain barrier and target brain metastases of NSCLC patients harboring sensitive EGFR mutations [10–13]. For patients with brain metastases, gefitinib as the first-line treatment attained an intracranial objective response rate (iORR) of 87.8%, while erlotinib as second-line treatment reached an iORR of 75% [14, 15]. Two retrospective analyses by Heon et al. [16, 17] reported that first-line TKI gefitinib/erlotinib treatment for EGFR mutant advanced NSCLC patients resulted in lower rates of CNS progression compared with first-line chemotherapy. These results indicated that gefitinib and erlotinib might have an effective role in prevention and treatment of CNS metastases in NSCLS patients harboring sensitive EGFR mutations. However, there is currently no definitive conclusion regarding comparative effectiveness between the two first generation TKIs gefitinib and erlotinib in the prevention and treatment of brain metastases in NSCLS with EGFR mutations. Erlotinib might be more effective in the prevention and treatment for brain metastases than gefitinib since the concentration of erlotinib in the cerebro-spinal fluid (CSF) reaches higher levels than that of gefitinib [18].

To our knowledge, there are no reports that directly compare erlotinib with gefitinib in preventing and controlling brain metastases in NSCLC patients harboring EGFR-sensitive mutations. The aim of this retrospective study is to analyze the prevention and control of brain metastases in a cohort of EGFR mutant NSCLC patients initially treated with erlotinib in comparison to that in a cohort initially treated with gefitinib.

## Methods

### Study design and patients

The medical records of advanced NSCLC patients were identified through a query of patient information for subjects prospectively enrolled in the patient information system of the gefitinib and erlotinib charity project of the Cancer Foundation of China (CFC) and were distributed across Daping Hospital, Xinqiao Hospital and Southwest Hospital of Third Military Medical University as well as First Affiliated Hospital of Chongqing Medical University. The CFC required patients to be examined for disease progression in the body and head every 2 months. Specifically, neurological progression was defined as having evidence of progression of brain metastasis (more than 20% extension of the longest diameter in MRI) or evidence of new intracranial metastases. All patients signed the informed consent, facilitating the collection of baseline clinical features, tumor pathologic types and clinical prognostic information. The research protocol of current study has been approved by the ethics committees of Daping Hospital, Xinqiao Hospital and Southwest Hospital of Third Military Medical University as well as First Affiliated Hospital of Chongqing Medical University.

NSCLC patients with EGFR-sensitizing mutations and stage IV or systemic recurrent stage I-IIIB disease were eligible for this study. The enrolled patients were treated with gefitinib at 250 mg/day or erlotinib at 150 mg/day as the first-line therapy. Stage I-IIIA NSCLC patients who had previously undergone definitive treatment and subsequently relapsed were enrolled. Patients treated with EGFR-TKIs after disease relapse from previous systematic treatments, including neoadjuvant chemotherapy, adjuvant chemotherapy and definitive chemoradiotherapy were also enrolled. However, to avoid the interference of previous treatments, enrolled subjects must finish those previous treatments at over 12 months prior to EGFR-TKIs treatment. From January 1, 2009 to December 31, 2013, advanced NSCLC patients who received erlotinib or gefitinib treatment and were followed up for more than 1 year were included in this analysis.

All patients took erlotinib or gefitinib at the discretion of the treating providers and underwent brain imaging on first diagnosis of NSCLC and/or at the recognition of advanced disease. Follow-up brain imaging examinations were done once every two months or as decided by the doctors based on CNS symptoms/signs suggestive of CNS involvement. Brain lesions were generally evaluated by MRI (magnetic resonance imaging). Contrast-enhanced computed tomography (CT) was applied instead of an MRI in some patients. CNS metastases included parenchymal brain metastases and radiographically diagnosed leptomeningeal disease. The progression incidence and time to development of brain and leptomeningeal metastases from the start of TKI treatment were collected.

### Mutation analysis

Consistent with previous methods using the ADx-ARMS EGFR Mutation Test Kit (AmoyDx), the tumor specimens were detected using the amplification refractory mutation system (ARMS) that can detect a total of 29 EGFR gene mutations [19]. In this study, the following EGFR mutations were defined as sensitive mutations: deletion or deletion-insertion of exon 19, point mutations of L858R, L861Q in exon 21, and missense point mutations of G719(G719S or G719C) in exon 18.

### Statistical analysis

For all patients, medical records were retrospectively reviewed for clinicopathological information and data. Tumor histology was classified according to the WHO 2004 standard [20]. The distribution of patient baseline characteristics between the treatment groups were analyzed with the Wilcoxon rank-sum test or the Fisher exact test. The cumulative incidence curve was used for the evaluation of cumulative risk of CNS progression with the Chi-square test. The Kaplan–Meier method was used for assessing nTTP and PFS. The progression time was calculated from the first day of EGFR-TKI treatment of advanced NSCLC to the progression of disease or death. If the patient did not die or showed no progression at the final follow-up, the results were considered as censored data. The log rank test was used for comparing the survival curves and CNS progression curves. The SPSS version 20.0.0 was used for statistical analysis and all *P* values were based on two-sided hypothesis testing.

## Results

### Clinical characteristics of patients

Between January 1, 2009, and December 31, 2013, 358 NSCLC patients with EGFR mutations from the CFC database were screened. Two hundred and seventy-nine patients with stage IV disease or relapsed metastatic NSCLC harboring sensitizing EGFR mutations were included in the final analysis and were treated with either gefitinib (*n* = 171) or erlotinib (*n* = 108) as their initial systemic therapy. Another 79 patients with EGFR-sensitizing mutations were excluded from the study, including 49 patients with less than 1 year of follow-up and 30 patients who received gefitinib/erlotinib treatment for recurrent lesions less than 12 months after completing neoadjuvant/adjuvant chemotherapy. The demographics and disease characteristics of the included patients were summarized (Table 1). The median age of the study cohort was 58 years (range, 32–84 years), with no significant difference between the two groups (*p* = 0.343). About 70% of patients were stage IV, 90% had adenocarcinoma histology, and 65% were never smokers at their initial diagnosis of NSCLC. The age, gender, smoking history, and disease characteristics were balanced between erlotinib and gefitinib cohorts except there were more patients with brain metastases in the erlotinib cohort than in the gefitinib cohort (22.2% vs 12.9%, *p* = 0.047) before EGFR-TKI first line treatment. In the erlotinib group, 14/24 patients were treated with erlotinib concurrently with whole-brain radiotherapy (WBRT) (4000 cGy/20f/4 W); 4/24 patients received stereotactic radiotherapy following WBRT; 2/24 patients with solitary brain metastases received WBRT after surgical resection. In the gefitinib group, 11/22 patients were treated with gefitinib concurrently with WBRT; 3/22 patients with solitary brain metastases underwent surgical resection, followed by WBRT in 1 patient; 5/22 patients received stereotactic radiotherapy after WBRT. The 7 remaining patients (erlotinib: 4; gefitinib: 3) had ≥ 4 brain metastasis foci, were asymptomatic, and received no localized CNS therapy.

**Table 1:** Clinical features of patients

Items	Erlotinib Group (*N* = 108)	Gefinitib Group (*N* = 171)	*P* value
Age, *y* (%)			0.719
≥ 60	45 (41.7%)	75 (43.9%)	
< 60	63 (58.3%)	96 (56.1%)	
Gender, *n* (%)			0.343
Male	53 (49.1%)	74 (43.3%)	
Female	55 (50.9%)	97 (56.7%)	
Smoking history ^a^, *n* (%)			0.359
Never smoking	70 (64.8%)	120 (70.2%)	
Now/once smoking	38 (35.2%)	51 (29.8%)	
ECOG PS score, *n* (%)			0.491
0-1	106 (98.1%)	165 (96.5%)	
2	2 (1.9%)	6 (3.5%)	
Pathological type, *n* (%)			1.000
Adenocarcinoma	98 (90.7%)	156 (91.2%)	
Non adenocarcinoma	10 (9.3%)	15 (8.8%)	
Cancer staging^b^, *n* (%)			0.088
Recurrent type ^c^	18 (16.7%)	44 (25.7%)	
IIIb stage	7 (6.5%)	17 (9.9%)	
IV stage	83 (76.9%)	110 (64.3%)	
Cerebral metastasis before EGFR-TKIs use, n (%)	24 (22.2%)	22 (12.9%)	0.047
Previous treatment of cerebral metastasis, No.			—
WBRT + TKI	14	11	
Surgery + WBRT	2	3 (1)	
WBRT + SRS	4	5	
None	4	3	
Cerebral metastasis number before EGFR-TKIs use, *n*			—
1	2	3	
2-3	4	5	
≥ 4	18	14	

^a^ Present smoker was defined as someone who had smoked more than 100 cigarettes in their lifetime but either currently being smoking or stopped smoking less than 1 year ago; former smoker was defined as someone who had smoked more than 100 cigarettes in their lifetime and stopped smoking at least 1 year ago; non-smoker was defined as having either smoked 100 or fewer cigarettes in their lifetime or had never smoked cigarettes

^b^ American Joint Committee on Cancer staging system 7th edition

^c^ Patients with stage I-IIIa NSCLC with systemic recurrence following definitive therapy that included surgical resection or radiotherapy

The EGFR mutation status of all patients was suitable for this analysis (Table 2). The EGFR mutations were detected in pretreatment tissue specimens (surgical specimens, puncture specimens or fiber bronchoscopic specimens) in 268/279 patients, whereas malignant pleural effusion cytology specimens were tested in 11/279 patients following treatment with an EGFR-TKI. The proportion of the classical sensitive mutations (deletion or deletion- insertion mutations of exon 19, L858R point mutation of exon 21) was similar in the two groups. In the gefitinib group, 1 patient with a G719A^b^ mutation of exon 18 was combined with a deletion mutation of exon 19; another patient with a G719A^b^ mutation of exon 18 was combined with a L858R point mutation of exon 21. No T790M primary drug resistance mutation of exon 20 was found.

**Table 2:** EGFR Gene mutation status

Exons	Mutation type	Frequency No. (%)^a^	
		Erlotinib Group	Gefinitib Group
18	G719A^b^	1 (0.9%)	2 (1.2%)
	G719S	0 (0)	1 (0.6%)
	G719C	1(0.9%)	1 (0.6%)
19^c^	Del	43 (39.8%)	84 (49.1%)
	Delins	6 (5.6%)	13 (7.6%)
20	T790M	0 (0)	0 (0)
	S768I	0 (0)	0 (0)
21^d^	L858R	47 (43.5%)	59 (34.5%)
	L861Q	10 (9.3%)	13 (7.6%)

*Abbreviations: del* stands for deletion; *delins* stands for deletion-insertion

^a^ Two patients had double mutation

^b^ One patient had G719A and 19 del; one patient had G719A and L858R

^c^ Mutation of exon 21 in Erlotinib Group and Gefinitib Group (45.4% vs 56.7%, *P* = 0.067)

^d^ Mutation of exon 19 in Erlotinib Group and Gefinitib Group (52.8% vs 42.1%, *P* = 0.086)

### Disease progression pattern

Up to the latest analysis time point (December 31, 2014), 171 surviving patients (erlotinib: 73; gefitinib: 98) had a median follow-up of 22 months (range, 3–98 months), with no significant difference between the two cohorts. As of the data cutoff point, 37% of patients (40/108) were continuing to receive their first-line erlotinib therapy and 36.1% of patients (39/108) switched to chemotherapy in the erlotinib group; while 35.7% of patients (61/171) were continuing to receive first-line gefitinib and 1.8% of patients (3/171) changed to erlotinib and 31.0% of patients (53/171) changed to chemotherapy. During the EGFR-TKI treatment, 26.9% of patients (29/108) died in the erlotinib group and 31.6% of patients (54/171) died in the gefitinib group.

CNS progression occurred in 18.5% of patients (20/108) in the erlotinib group and 23.4% of patients (40/171) in the gefitinib group. Of the 60 patients who developed CNS progression, 18 patients had previously received treatment for brain metastases (6 in erlotinib; 12 in gefitinib). Leptomeningeal metastasis occurred in 4 patients (3.7%) in the erlotinib group and 6 patients (3.5%) in the gefitinib group, and 7 of these 12 patients had synchronous brain metastases at the time of diagnosis of leptomeningeal involvement. In the erlotinib group, 15 patients had the CNS as the primary foci of metastasis, and the only site of first progression in 10 of these 15 patients. The respective numbers for the gefitinib group were 16 patients as the initial site of progression and 13 as the only site of first progression.

**Fig. 1:**
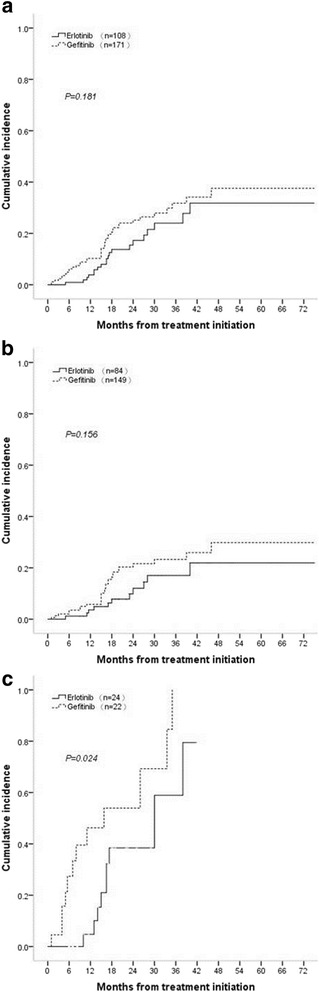
Cumulative incidence of CNS progression (**a**) in all eligible patients (*n* = 279); **b** patients without cerebral metastasis prior to EGFR-TKIs first-line treatment; **c** patients cerebral metastasis prior to EGFR-TKIs first-line treatment

The cumulative incidence curves of CNS progression for each group are shown in Fig. 1. The incidence rates of cumulative CNS progression at 6-, 12- and 18-months were 0.9, 3.7 and 12.0% in the erlotinib group and 5.8, 9.4 and 17.0% in the gefitinib group (*P* = 0.181, Fig. 1a). The median nTTP was significantly longer in the erlotinib group than in the gefitinib group (24 months vs 16 months, *p* = 0.014). The HR of CNS progression for upfront erlotinib versus gefitinib was 0.695 [95% confidence interval (CI), 0.406–1.190], suggesting a risk reduction of 30.5% although without statistical significance. For those 233 patients without preexisting brain metastases prior to EGFR-TKI first line treatment, the median nTTP was 18 months and 16 months respectively in the erlotinib group and gefitinib group (*p* = 0.392) and the 6-, 12- and 18-month cumulative rates of CNS progression were 1.2, 3.6, and 7.1% in the erlotinib group compared with corresponding rates of 3.4, 5.4, and 13.4% in the gefitinib group (*p* = 0.156, Fig. 1b). However, for those patients with preexisting brain metastases before EGFR-TKI treatment, erlotinib first line treatment could significantly extend the median nTTP in comparison to gefitinib (30 months vs 15.8 months, *p* = 0.024, Fig. 1c).

**Fig. 2:**
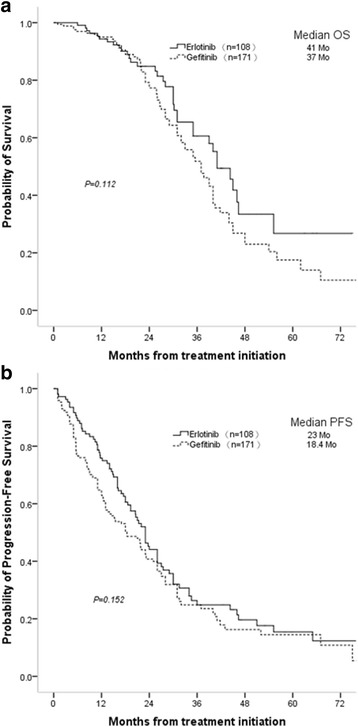
Overall survival (**a**) and progression-free survival (**b**) in all patients (*n* = 279). OS: overall survival; PFS: progression–free survival

From the start of erlotinib and gefitinib treatment, the median OS (mOS) was 41 months and 37 months respectively (*p* = 0.112, Fig. 2a), and the mPFS was 23 months and 18.4 months respectively (*P* = 0.152, Fig. 2b). From the diagnosis of CNS progression, the mOS was 16 months and 12.6 months in the erlotinib group and gefitinib group (*p* = 0.793). CNS progression increased the death risk in comparison to no CNS progression (HR = 3.73, *p* = 0.000) in all eligible patients; interestingly, the HR for death was 2.37 (*p* = 0.023) in the erlotinib cohort and 5.46 (*p* = 0.000) in the gefitinib cohort.

## Discussion

In the study, the effects of erlotinib versus gefitinib as first-line treatment on the risk of CNS progression in advanced NSCLC patients with EGFR mutations was retrospectively analyzed. nTTP was significantly improved in preexisting brain metastases patients with first line erlotinib treatment compared with gefitinib (30 months vs 15.8 months, *P* = 0.024). Although the cumulative incidence of CNS progression at 6-, 12-, and 18-months was not significantly different (*P* = 0.181) in the erlotinib group compared with the gefitinib group, the HR of CNS progression for upfront erlotinib versus gefitinib was 0.695 (95% confidence interval [CI], 0.406–1.190), suggesting a CNS progression risk reduction of 30.5% in the erlotinib group. To our knowledge, this is the first retrospective study examining the impact of initial therapy of two first generation EGFR-TKIs on the prevention and control of CNS progression in EGFR–mutant NSCLC patients, which could provide an important basis for standardized management of EGFR-TKI therapy for CNS progression of NSCLC with EGFR-sensitizing mutations.

The two first generation EGFR-TKIs have not yet been demonstrated to be the therapeutic choice for treatment of patients with CNS progression carrying EGFR mutations. Heon et al. reported in retrospective studies that CNS progression in advanced NSCLC patients treated with chemotherapy was more than 40% and that the first generation TKIs (erlotinib or gefitinib) could significantly decrease CNS progression to about 30% [16, 17]. However, there is no data to compare these two first generation TKIs with respect to CNS progression in advanced EGFR mutant NSCLC patients. In the present study, *EGFR*-mutant advanced NSCLC patients without preexisting brain metastases showed no significant difference in the cumulative rates of CNS progression (*P* = 0.156) and the median nTTP (18 months vs 16 months, *P* = 0.392) between the erlotinib group and gefitinib group. However, for the advanced EGFR mutant NSCLC patients with prior CNS involvement, the time to occurrence of CNS progression was significantly prolonged after first-line erlotinib compared with gefitinib (30.0 months vs 15.8 months, *P* = 0.024), indicating the greater potential of erlotinib at slowing development of established CNS metastases from NSCLC than gefitinib. Our observations highlight the greater effectiveness and importance of controlling preexisting brain metastasis in *EGFR*-mutant advanced NSCLC patients by erlotinib treatment.

Erlotinib significantly prolonged nTTP compared with gefitinib in the treatment of CNS metastases in NSCLC patients with preexisting brain metastasis. The related mechanism explaining such a result remains uncertain. However, data from several studies allows us to infer some possible hypotheses. The higher penetration of erlotinib through the brain blood barrier might explain why erlotinib treatment could prolong nTTP in preexisting brain metastasis. In phase I trials of targeted therapy, erlotinib and gefitinib delivered at standard daily dosing is the maximum tolerated dose or optimal biological dose, respectively, resulting in a plasma exposure concentration of erlotinib 7 times greater than that of gefitinib [21, 22]. In addition, when EGFR-TKI serum concentrations were associated with CSF concentrations, the erlotinib CSF concentration was significantly higher than that of gefitinib (66.9 ± 39.0 nM vs. 8.2 ± 4.3 nM, *P* = 0.0008) [11, 18]. Another experimental study from Carey and Li et al. [19, 23] using kinetic analysis showed that erlotinib has a stronger antitumor effect than gefitinib when using the conventional recommended dose. Furthermore, a report performed by Masuda et al. [24] showed clinical improvements following the change to erlotinib therapy in lung adenocarcinoma patients with EGFR mutations who developed leptomeningeal metastases during gefitinib therapy. Our observations highlight the importance of elucidating the potential CNS efficacy of erlotinib.

For CNS progression in NSCLC patients without prior CNS metastases, no significant difference was found between erlotinib and gefitinib treatment. Notably, EGFR mutation status as a poor prognostic factor for the risk of brain metastasis in NSCLC has previously been demonstrated. In a retrospective trial of 314 lung adenocarcinoma patients with EGFR mutations, the multivariate model analysis showed a strong association between EGFR mutation status and brain metastasis (adjusted odds ratio = 3.83, 95% CI: 1.72-8.55, *P* = 0.001) [4]. While resistance to continued EGFR inhibition is common, acquired systemic resistance through the selection of resistance mutations or amplification of other oncogenes is usually detected after 6 to 12 months of therapy [25, 26]. Previous studies have shown that CNS penetration of erlotinib and gefitinib at standard daily dosing is limited [18, 27]. One additional recognized mechanism of pharmacokinetic resistance, a poor CSF-to-plasma ratio, occurs in patients who continue to have systemic disease controlled with gefitinib or erlotinib but display progression or new-onset CNS disease. Further clinical cohort studies need to be performed to examine this further. To achieve adequate CNS concentrations, the dosing schedule may also be important, for example pulsatile dosing schedules [28], concentrations of gefitinib [29] or designing new drugs (such as AZD3579) [30]. Additional possibility is another first generation EGFR-TKI, afatinib, which is much less commonly used in China due to the limited availability and cost-effect reasons. Martin Schuler et al. [31] showed afatinib significantly improved the ORR versus chemotherapy in patients with NSCLC and asymptomatic CNS metastases. Their findings suggested the clinical activity of afatinib in EGFR mutation–positive NSCLC patients with brain metastases. However, to our knowledge, there are no study comparing the effectiveness on the NSCLC patients with CNS metastases of three first generation EGFR-TKIs in parallel.

Another concern when considering to put this strategy forward in clinical practice would be the neuro recognition function or the quality of life (QoL). As both the neuro recognition function and QoL measurements require special methodologies, these data are missing in our cohort. However, previous report showed that grade 3/4 adverse event rates were similar (70.0%) in WBRT with or without erlotinib, except for rash was higher and fatigue was higher. No statistically significant quality of life differences was found [30]. But, again, the neuro recognition function were not reported. With the survival improvement of NSCLC patients with CNS metastases, additional studies focusing on these long term effects will be in need.

Our findings are limited to those of any retrospective analysis. First, the number of brain metastases patients at the time of diagnosis was not balanced in the erlotinib group and gefitinib group. However, the therapeutic measures for preexisting CNS metastasis were well balanced between the two cohorts and the results of nTTP for patients with preexisting CNS metastasis should be valid. Second, the CSF-to-plasma concentration ratios were not detected in this study. In addition, we did not evaluate other clinically important genetic changes besides EGFR mutations, for example, KRAS mutation, c-Met amplification, or the echinoderm microtubule-associated protein-like 4 anaplastic lymphoma kinase (ALK) translocation. Thus, we were unable to evaluate possible interactions between these genes and CNS progression. Despite these limitations, our study was valuable in view of the new insights that erlotinib showed significantly prolonged nTTP compared with gefitinib in the treatment of CNS metastases in NSCLC patients with preexisting brain metastasis, and that no difference exists in treatment of micrometastatic CNS disease and CNS progression in NSCLC patients without prior CNS metastases.

At present, there is still a lack of effective drugs for brain metastasis of NSCLC. Our findings provide a rationale for physicians to use erlotinib for the treatment of CNS progression in EGFR mutant NSCLC. Our findings need to be further confirmed in prospective studies with a larger sample size. Due to some evidence of the beneficial effect and low toxicity of erlotinib [15], the clinical use of EGFR inhibitors concurrently with radiation therapy is currently being investigated in several clinical trials including the NCT 01887795 trial.

## Conclusions

In conclusion, our results suggest significantly prolonged nTTP of CNS metastases in EGFR-mutant NSCLC patients with preexisting brain metastasis initially treated with erlotinib compared with upfront gefitinib. If validated, our findings suggest that erlotinib might be more effective at delaying CNS metastases from NSCLC in patients with sensitizing EGFR mutations. As EGFR mutant NSCLC was identified as a sensitive molecular subtype for EGFR-TKI inhibition, there is a need to conduct carefully designed trials with specific CNS endpoints to evaluate whether the candidates for targeted therapy of CNS penetration can treat and/or prevent the occurrence or recurrence of established CNS metastases.

## Additional file

Additional file 1: The raw clinical data of current study. (XLSX 57 kb)

## Abbreviations

ALK: Anaplastic lymphoma kinase
ARMS: Amplification refractory mutation system
CFC: Cancer Foundation of China
CI: Confidence interval
CSF: Cerebro-spinal fluid
CT: Computed tomography
CNS: Central nervous system
EGFR: Epidermal growth factor receptor
IORR: Objective response rate
MOS: Median OS
MPFS: Median progression-free survival
NSCLC: Non-small cell lung cancer
NTTP: Neurological progression
ORR: Objective response rate
OS: Overall survival
WBRT: Whole-brain radiotherapy
